# Genetic variants associated with reduced body size in indigenous chickens of Eritrea: A genome-wide association study

**DOI:** 10.1016/j.psj.2026.107502

**Published:** 2026-07-27

**Authors:** Hortuma A. Habteslasie, Francesco Perini, Kiplangat Ngeno, Daniele Colombi, Emiliano Lasagna, Tadelle Dessie, Alexander Kahi

**Affiliations:** a Agricultural Extension Department (AED), Ministry of Agriculture, Government of the State of Eritrea, Asmara, Eritrea; b Animal Breeding and Genomic Group, Department of Animal Science, Egerton University, Egerton, Njoro, Kenya; c Department of Agricultural, Food and Environmental Sciences, University of Perugia, Borgo XX Giugno, 74, Perugia, 06121, Italy; d Department of Agriculture, Animal Science and Natural Resources, Moi university, Eldoret, Kenya; e International Livestock Research Institute (ILRI), P.O. Box 5689, Addis Ababa, Ethiopia

**Keywords:** Bantam-like phenotype, Indigenous chicken, Growth regulation, Resource-limited system, Environmental adaptation

## Abstract

Indigenous chickens in Eritrea are raised mainly in low-input village systems and show marked variation in body size, including reduced body size phenotype, a trait associated with reduced feed consumption, thermotolerance, disease resistance, and adaptability to resource-limited environments. This study aimed to identify genomic regions associated with reduced body size using a genome-wide association study (GWAS) in Eritrean chicken ecotypes. Blood samples from 384 ICs across 16 ecotypes (21 to 24 birds per ecotype) were genotyped using DArTseq technology. Three putative short-bodied ecotypes, Barentu (BAR), Foro (FORO), and Gogne (GOG), were compared with five heavier control ecotypes: Fshe-Mrara (FM), Adi-Tekeliezan (ADTEK), Emni-Haili (HAYL), Dekemhare (DEKE), and Adikeyh (KEIH). After filtering for individual missingness, marker call rate, and minor allele frequency, 141 birds and 42,356 SNP remained. Population structure was evaluated by principal component analysis and ADMIXTURE. Exploratory case-control GWAS were conducted for each putative reduced-body-size ecotype and for the 3 ecotypes combined. Complementary quantitative-trait GWAS used body weight, body length, back length, and shank length was carried out. Linear mixed models implemented in GEMMA included sex as a fixed effect and genomic kinship as a random effect. The first 2 principal components showed extensive ancestry sharing, although GOG was more differentiated. Ecotype-specific analyses identified significant regions near *CDK6, BDNF, FOXP1, TNS3*, and *LGR4* in FORO; *EIF2AK2, NANP, MDH1, UGP2* and *FGF13* in GOG; and *HMGA2, IGF2, BRSK1* and *SUCNR1* in BAR.

The combined analysis showed no genome-wide significant variants but revealed suggestive signals near *ASAP1, EIF2AK2*, and *FOXP1*, indicating a polygenic and ecotype-specific architecture. Overlapping candidate genes across analyses included *TH* (common to all) and *EIF2AK2* (present in all ecotypes except FORO). Quantitative-trait analyses detected a single significant SNP within *CACNB4* for back length, but none for the other traits. Overall, reduced body size in Eritrean IC appears to be a complex trait controlled by multiple loci, involving both conserved growth regulators and genes related to metabolic efficiency and environmental adaptation. These findings provide a genomic basis for future breeding strategies to improve productivity, resilience and sustainability of village poultry production systems in resource-limited regions of Eritrea.

## Introduction

Indigenous chickens (IC) play a key role in rural production systems in sub-Saharan Africa, where they contribute to food security and household income under low-input conditions (FAO, 2015; Mottet and Tempio, 2017). In Eritrea, as in many African countries, these populations are predominantly raised in village systems and exhibit high levels of phenotypic and genetic diversity (FAOSTAT, 2023; Habteslasie, 2018; Singh et al., 2023). Despite their adaptability to harsh environments, IC are generally characterized by low productivity, mainly due to slow growth and reduced body size (Milkias et al., 2019).

Among the phenotypic traits observed in these populations, reduced body size represents a biologically and economically relevant feature. Chickens with smaller body size are often associated with improved feed efficiency, thermotolerance, and resilience under environmental stress, making them particularly suitable for low-input production systems (Ngeno et al., 2014; Yeasmin and Howlider, 2013). Moreover, this phenotype is valued for high egg-laying performance an fertility (Missohou et al., 2003; Njenga, 2005; Uddin et al., 2014), disease resistance (Abdi-Soojeede, 2022), predator defence capability, and resilience under challenging conditions, including limited access to feed and water, and high temperatures (Missohou et al., 2003), alongside strong maternal instinct (Mahoro et al., 2017). More broadly, the assumption that bigger is always better should be evaluated against the production-system setting, as smaller body size may contribute to sustainability under resource-limited conditions.

These phenotypes have been widely reported in tropical and subtropical regions, where they are maintained through both natural and farmer-driven selection. As an eco-friendly genotype, reduced-body-size chickens, traditionally described as dwarf and/or bantam-like chickens, occur widely across the Global South. They are known locally as Dooro Jarray in Somalia (Abdi-Soojeede, 2022), Betwil in Sudan (Mohammed et al., 2005; Yousif and Eltayeb, 2011), Jarso in Ethiopia (Reta, 2009), Degueredje in Burkina Faso (Ouédraogo et al., 2023), Dourgou in Niger (Hassan et al., 2020), Kambwata/ Simboti in Malawi (FAO, 2003), Chineya in Zimbabwe (Wilson, 2021), Tswana/Dwarf in Botswana (Machete, 2023), Nicobari in India (Haunshi et al., 2025), and Moarn Cher in Cambodia (Menghak and Chagunda, 2022), among others.

These chickens represent a valuable opportunity for poverty alleviation due to their adaptability to free scavenging and rapid movement in the wild (Abdi-Soojeede, 2022; Wilson, 2021). In Eritrea, information on the genetic determinants of body size variation in ICs remains limited. Although dwarf-like phenotypes have been reported in local ecotypes, their genetic basis has not been characterized, and phenotypic assessment alone may not reliably distinguish true genetic dwarfism from environmentally induced size variation (Perini et al., 2023). This knowledge gap limits the potential use of these populations in targeted breeding strategies.

Genome-wide association studies (GWAS) provide a powerful approach to identify genetic variants associated with complex traits such as body size, enabling the detection of candidate genes and biological pathways underlying phenotypic variation (Wu et al., 2021). Applying GWAS to indigenous populations can reveal both conserved growth regulators and novel adaptive variants linked to environmental resilience.

Therefore, the aim of this study was to investigate the genetic architecture of reduced body size in Eritrean ICs using a genome-wide association approach. By comparing ecotypes with contrasting body weights, we sought to identify genomic regions, candidate genes, and biological pathways involved in growth regulation and adaptation to low-input production systems.

## Materials and methods

### Ethical statement

Ethical approval was not required for the current study. Blood samples were collected in compliance with the indications and regulations outlined in the ARRIVE guidelines (https://arriveguidelines.org), during routine health controls by the public veterinary service.

### Study population and phenotypes

The study was conducted in Eritrea and encompassed 16 IC ecotypes, representing approximately 24% of the national sub-regions, with elevations ranging from 21 to 2528 m above sea level. Ecotypes were selected based on the concentration of IC, minimal presence of exotic chicken strains, strategic importance, accessibility, and market connectivity (Habteslasie and Araya, 2018). In total, 384 IC (*Gallus gallus domesticus*), comprising 21-24 individuals per ecotype and approximately equal numbers of males and females, were sampled from the following ecotypes: Adi-Tekeliezan (ADTEK), Keren (KER), Hagaz (HAGAZ), Mensura (MEN), Barentu (BAR), Gogne (GOG), Tokombia (TOK), Shambqo (SHAM), Molqi (MOLQ), Emni-Haili (HAYL), Mai-Ayni (AYNI), Dekemhare (DEKE), Adikeyh (KEIH), Mai-Mne (MNE), Foro (FORO), Fshe-Mrara (FM).

**Fig. 1:**
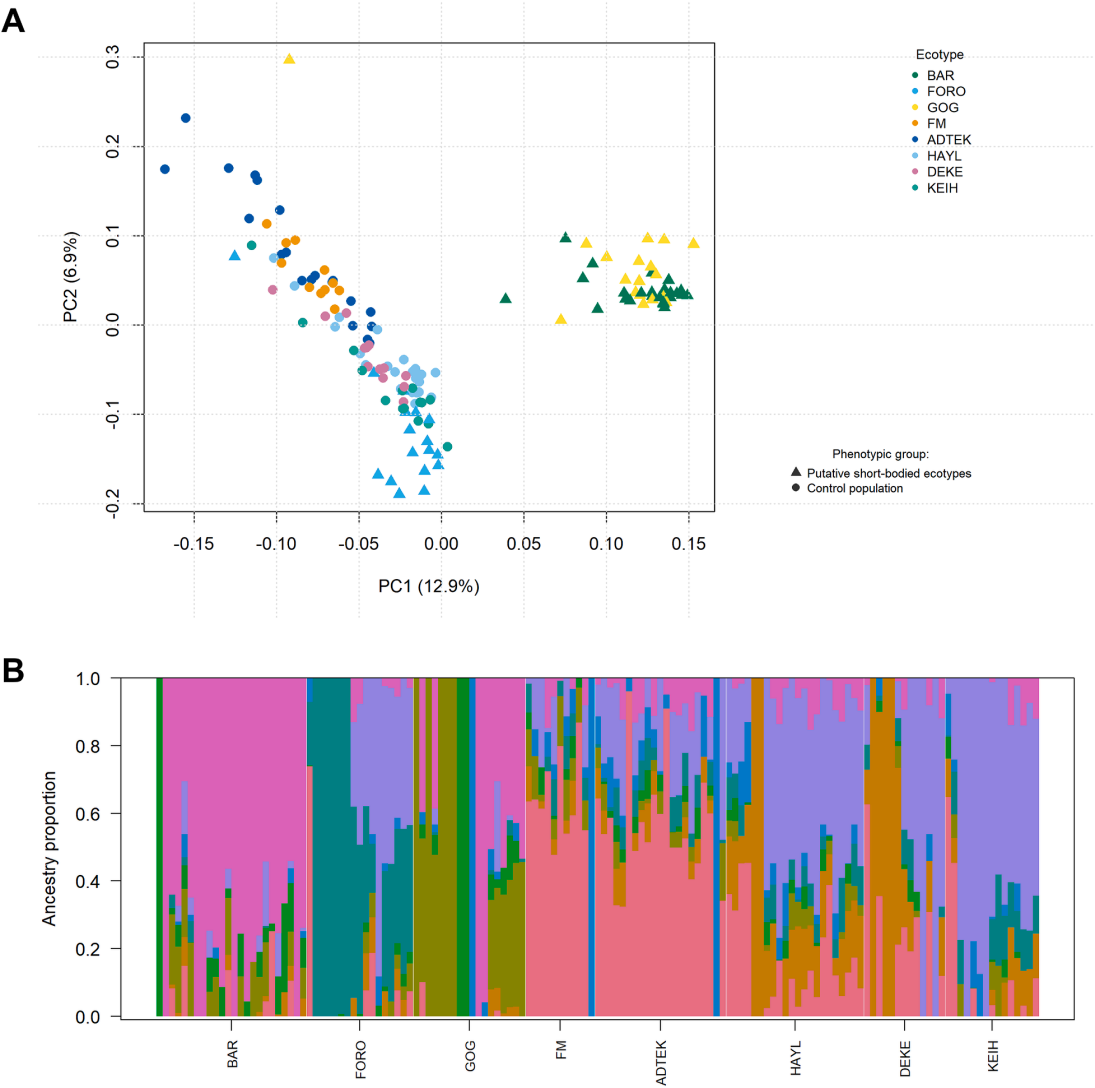
Box plots of phenotypic measurements across Eritrean chicken ecotypes. Body weight values in kg; body length, back length, and shank length in cm**.**

The birds were raised under traditional village production systems without standardized feeding regimes. Live body weight, body length, back length, and shank length were recorded following FAO phenotypic-characterization guidance (Perini et al., 2020; Bekalu et al., 2023; FAO, 2012). Accordingly, the three ecotypes with the lowest mean body weight and body-size measurements, BAR, FORO, and GOG, were classified as putative short-bodied cases, whereas FM, ADTEK, HAYL, DEKE, and KEIH, which had the highest mean body weight and larger linear measurements, were selected as control (Fig. 1). This classification was based exclusively on morphometric measurements and was not intended to represent clinically, endocrinologically, or molecularly confirmed genetic dwarfism.

### DNA extraction, genotyping, and quality control

Blood was drawn from the wing vein using syringes and preserved on FTA Whatman TM cards (Qiagen), with a maximum volume of 125 μl per spot, in line with Krambrich et al. (2022). DNA isolation and genotyping were performed at SEQART Africa laboratories within the BecA hub at ILRI, Nairobi, using GBS DArTseq technology to identify SNP variation (Kimwemwe et al., 2023; Mujyambere et al., 2024). Genomic DNA was extracted from FTA card punches using a standard protocol for dried blood spots (Krambrich et al., 2022). DNA extraction via the Nucleomag kit yielded concentrations from 50 to 100 ng/ μl (Mujyambere et al., 2024; Wahome, 2023). DNA quality and concentration were assessed via NanoDrop spectrophotometry and agarose gel electrophoresis. Genotyping was conducted using DArTseq, which employs restriction enzyme digestion to reduce genome complexity, followed by sequencing on the Illumina HiSeq 2500 platform, in line the protocols of Wahome (2023), Mujyambere et al. (2024), and Adomako et al. (2024). SNP were aligned to the *Gallus gallus* GRCg6a reference genome (GCF_015476345.1) as suggested by Perini et al. (2024) for local genotypes. Only the eight ecotypes included in the association analyses were retained, yielding 179 individuals genotyped at 64,030 polymorphic SNP. Quality control of raw genotypes was conducted in PLINK v1.9 (Chang et al., 2015). Individuals with more than 10% missing genotypes (*–mind* 0.10), SNP with a call rate below 95% (*–geno* 0.05), and SNP with a minor allele frequency (MAF) below 0.05 (–maf 0.05) were excluded.

### Population structure

Principal Component Analysis (PCA) was performed in PLINK v1.9 (Purcell et al., 2007; Chang et al., 2015) using the filtered SNP dataset, and the first two principal components were visualized using R software. Individual ancestry proportions were inferred by applying the model-based clustering algorithm implemented in ADMIXTURE v1.3.0 at K = 8 (Alexander et al., 2009). The BITE R package was used to graphically represent the results (Milanesi et al., 2017). The most likely number of populations was estimated with the cross-validation procedure.

### GWAS analysis

GWAS compared the case and control population. Analyses were conducted for each putative short-bodied ecotypes individually, as well as for all putative short-bodied ecotypes combined, against the control group. Complementary quantitative-trait GWAS were conducted for body weight, body length, back length, and shank length using the same filtered genotype data. Association analyses were performed in GEMMA software v0.94.128 (Zhou and Stephens, 2012) using a linear mixed model. Sex was included as a fixed effect, and a genomic kinship matrix was fitted as a random effect to account for relatedness. The genome-wide significance threshold was set using the Bonferroni correction, with the p-value cut-off calculated as 0.05 divided by the number of tests performed, expressed as −log₁₀(0.05/number of variants) (Perini et al., 2025a). The genomic inflation factor λ was calculated as the ratio of the observed median chi-square statistic to the expected median under a chi-square distribution using the qqman package in R (Turner, 2018) in line with procedure implemented by Perini et al. (2025b).

Quantile-quantile (Q-Q) plots and the genomic inflation factor (λ) were used to evaluate model calibration and residual population structure (Devlin and Roeder, 1999; Turner, 2018). Only SNP meeting the genome-wide significance P-value threshold were retained for downstream analysis.

### Bioinformatics analysis

Significant SNP were annotated using the ‘GALLO’ package in R (Fonseca et al., 2020) to identify genes within a 0.1 Mb window. Overlap among positional candidate-gene lists from the three ecotype-specific analyses and the combined analysis was assessed using Venn diagrams generated with the InteractiVenn web tool (Heberle et al., 2015). Gene Ontology (GO) and Kyoto Encyclopaedia of Genes and Genomes (KEGG) analyses of predicted target genes were estimated using the Database for Annotation, Visualisation and Integrated Discovery (DAVID; https://david.ncifcrf.gov/) (Huang et al., 2009), and only pathways were assessed, applying Benjamini–Hochberg false discovery rate (FDR) correction, with significance set at FDR < 0.05.

## Results

### Genotype quality control

After quality control of the subset of 179 animals, 38 individuals with a genotype missing rate >10% were excluded. SNP with a genotype call rate <95% or a MAF <0.05 were also removed, excluding 21,674 markers. After quality control, the final dataset consisted of 141 individuals and 42,356 SNP that were retained for all downstream population structure and genome-wide association analyses.

### Population structure analysis

**Fig. 2:**
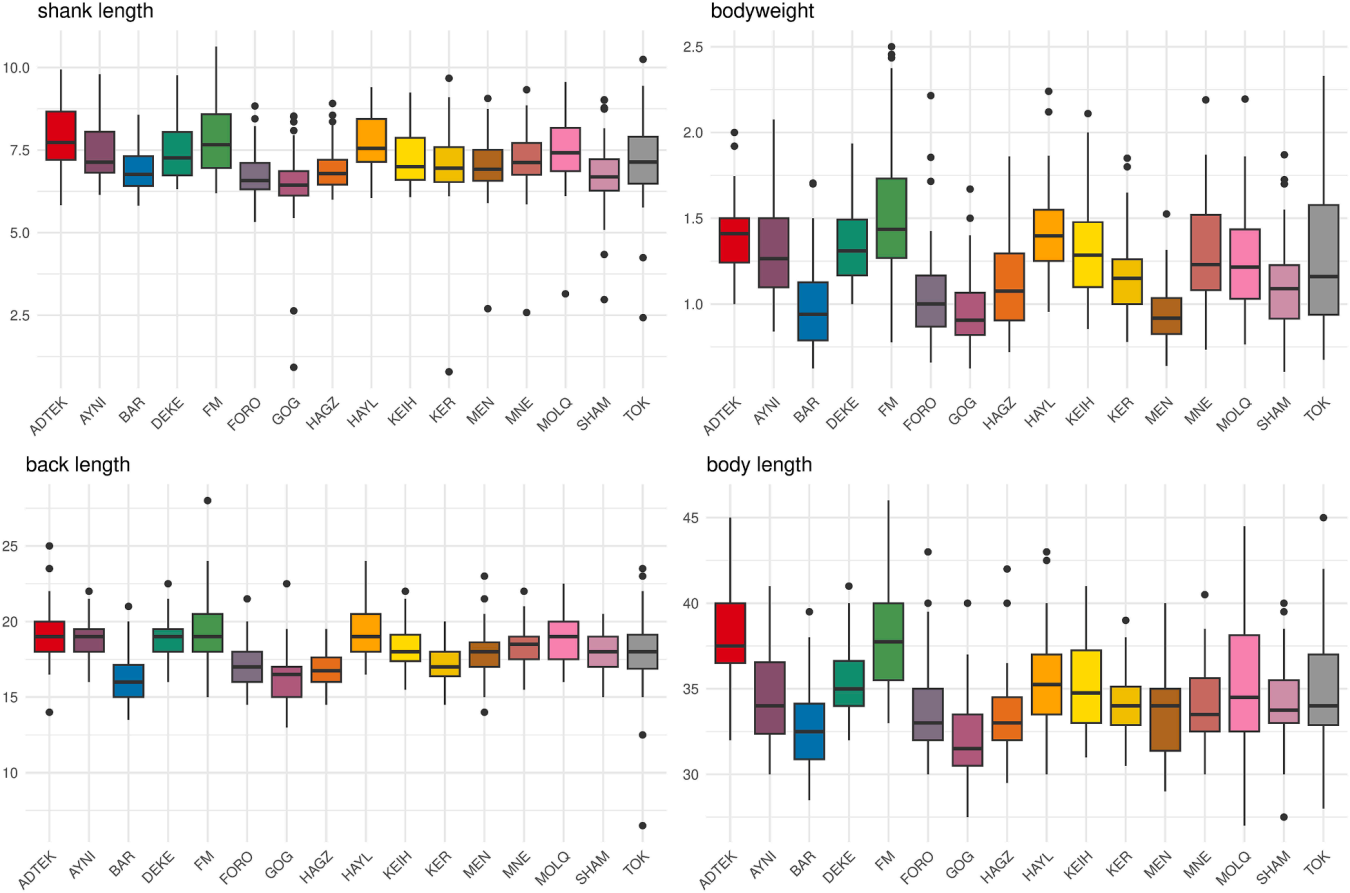
**(A)** Principal component analysis (PCA). Colours indicate ecotypes, whereas symbols distinguish putative short-bodied ecotypes (triangles) from control populations (circles). **(B)** ADMIXTURE analysis showing the inferred ancestry proportions of each individual. Each vertical bar represents one individual, and each colour corresponds to a different inferred ancestral component.

The first two principal components explained 13.0% and 6.9% of the total genomic variation, respectively (Fig. 2A). PCA revealed moderate genetic differentiation among ecotypes. The three putative reduced body size ecotypes did not cluster as a single homogeneous genetic group. BAR and FORO largely overlapped with several control ecotypes, whereas GOG formed a more distinct cluster along the first principal component. Likewise, the control ecotypes exhibited partial overlap, indicating the absence of clear genetic boundaries among most of the analysed populations.

ADMIXTURE analysis produced results consistent with the PCA (Fig. 2B). Individuals from all ecotypes showed varying proportions of shared ancestry components, with extensive admixture across populations. Although some ancestry components were more frequent within individual ecotypes, no ancestry profile was exclusive to either the putative short-bodied or the control populations. Overall, these analyses revealed substantial sharing of genetic ancestry among the eight village ecotypes included in the GWAS.

### Ecotype-specific case-control GWAS

**Fig. 3:**
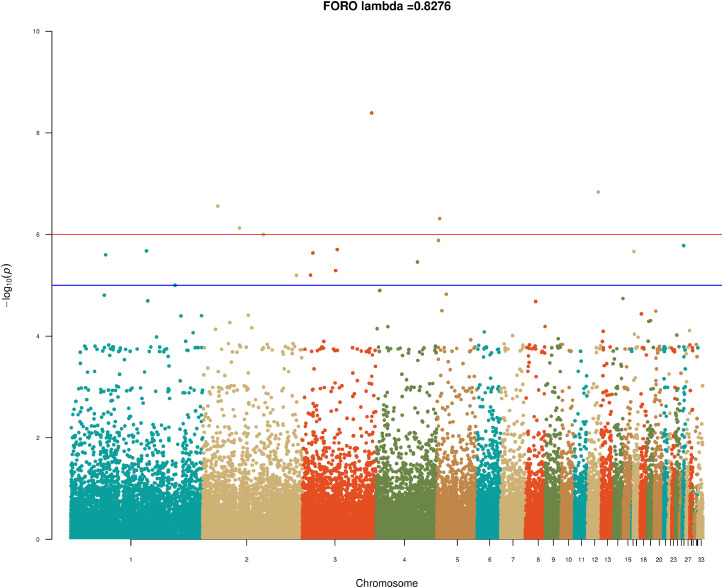
Manhattan plot of GWAS results for the FORO ecotype. Each point represents a SNP. The red and blue lines denote the genome-wide and suggestive significance thresholds, respectively.

**Fig. 4:**
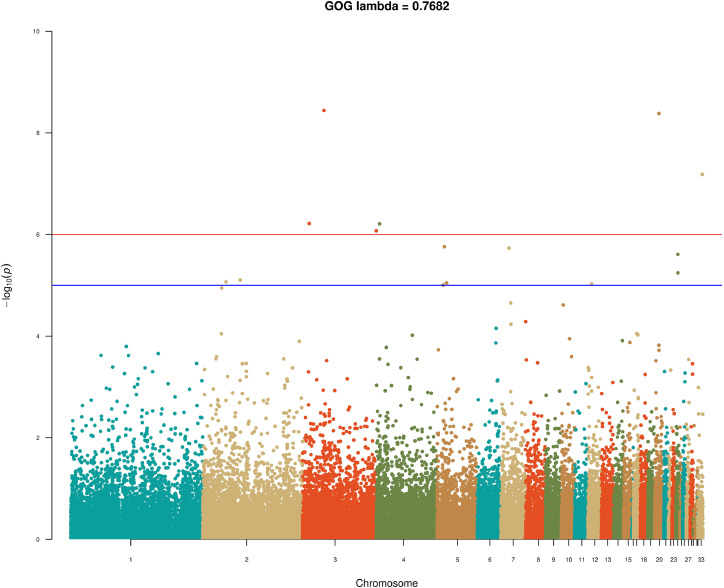
Manhattan plot of GWAS results for the GOG ecotype. The red and blue lines denote the genome-wide and suggestive significance thresholds, respectively.

**Fig. 5:**
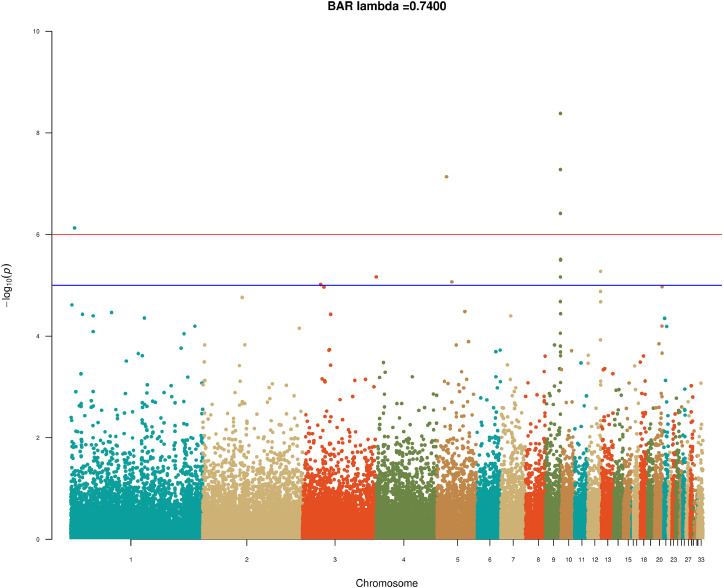
Manhattan plot of GWAS results for the BAR ecotype. Genome-wide and suggestive significance thresholds are shown by the red and blue lines, respectively.

The ecotype-specific GWAS identified genome-wide significant loci in each of the three putative reduced-body-size ecotypes (Fig. 3, Fig. 4, Fig. 5). The associated regions differed among ecotypes, consistent with genetic heterogeneity in the morphometric contrast.

### FORO ecotype

GWAS of the FORO ecotype revealed multiple SNP surpassing the genome-wide significance threshold (P < 5 × 10^−8^) on chromosomes 2, 3, 5, and 12 (Fig. 3). Genomic inflation factor of λ = 0.8276, indicates no evidence of inflation in the association statistics (Supplementary Fig. 1). The strongest association signals were located in close proximity to the genes *TNS3, CDK6, LGR4, BDNF*, and *FOXP1*. Functional annotation identified enrichment for regulation of monocyte differentiation, involving *CDK6* and *FOXP1*; predicted regulation of FOXP1 by gga-miR-9*-5p; CDK4/ CDK6* inhibition and oncogene-induced senescence pathways involving *CDK6;* and *NTRK2* signaling pathways involving *BDNF* (Table 1).

**Table 1:** Functional annotation of candidate genes from significant SNP regions in FORO ecotype.

Source	Term name	Term ID	Adjusted P-value	Intersections
GO:BP	regulation of monocyte differentiation	GO:0045655	0.02	*CDK6, FOXP1*
GO:BP	monocyte differentiation	GO:0030224	0.03	*CDK6, FOXP1*
miRNA	gga-miR-9-5p	MIRNA:gga-mir-9-5p	0.04	*FOXP1*
REAC	Drug-mediated inhibition of CDK4/CDK6 activity	REAC:R-GGA-9754119	0.04	*CDK6*
REAC	Activated NTRK2 signals through PLCG1	REAC:R-GGA-9026527	0.04	*BDNF*
REAC	Activated NTRK2 signals through FYN	REAC:R-GGA-9032500	0.04	*BDNF*
REAC	Activated NTRK2 signals through FRS2 and FRS3	REAC:R-GGA-9028731	0.04	*BDNF*
REAC	Oncogene Induced Senescence	REAC:R-GGA-2559585	0.04	*CDK6*
REAC	NTRK2 activates RAC1	REAC:R-GGA-9032759	0.04	*BDNF*

### GOG ecotype

In the GOG ecotype (Fig. 4), significant SNP were detected on chromosomes 3, 4, 20, and 33, with the peak on chromosome 3 showing −log₁₀(P) ≈ 8. The genomic inflation factor (λ = 0.7682) indicated minimal bias (Supplementary Fig. 2). Candidate genes mapped near significant regions included *EIF2AK2, NANP, GPATCH11, STRN, MDH1, UGP2, VPS54, PELI3, RECK, FGF13, CCNDBP1, EPB42, BANF1, POLA2,* and *CAPN1*. Enriched categories were dominated by amino-sugar, nucleotide sugar, carbohydrate, glycogen, and gluconeogenesis pathways involving *NANP, UGP2*, and *MDH1* (Table 2)*.*

**Table 2:** Functional annotation of candidate genes from significant SNP regions in GOG ecotype.

Source	Term name	Adjusted p-value	Intersections
KEGG	Amino sugar and nucleotide sugar metabolism	0.01	*NANP, UGP2*
KEGG	Biosynthesis of nucleotide sugars	0.01	*NANP, UGP2*
REAC	Carbohydrate metabolism	0.02	*MDH1*
REAC	Formation of the active cofactor, UDP-glucuronate	0.02	*UGP2*
REAC	Glycogen metabolism	0.02	*UGP2*
REAC	Glycogen synthesis	0.02	*UGP2*
REAC	Glucuronidation	0.02	*UGP2*
REAC	Gluconeogenesis	0.02	*MDH1*
GO:MF	UTP:glucose-1-phosphate uridylyltransferase activity	0.03	*UGP2*
GO:MF	UTP-monosaccharide-1-phosphate uridylyltransferase activity	0.03	*UGP2*
GO:MF	N-acylneuraminate-9-phosphatase activity	0.03	*NANP*
REAC	Metabolism	0.04	*MDH1*
REAC	Phase II - Conjugation of compounds	0.04	*UGP2*
REAC	Retrograde transport at the Trans-Golgi-Network	0.04	*VPS54*

### BAR ecotype

In the BAR ecotype (Fig. 5), five SNP exceeded the genome-wide significance threshold on chromosomes 1, 5, and 9. Significant SNP were located near the positional candidate genes *HMGA2, BRSK1, IGF2, SUCNR1*, and *AADAC*. The genomic inflation factor (λ = 0.7400) indicated no evidence of inflation in the association statistics (Supplementary Fig. 3). Functional enrichment identified biological processes related to carbohydrate and glucose homeostasis, protein secretion and extracellular localization and renin-angiotensin regulation, principally involving *BRSK1* and *SUCNR1* (Table 3).

**Table 3:** Functional annotation of candidate genes from significant SNP regions in BAR ecotype.

Source	Term name	Term ID	Adjusted p-value	Intersections
GO:BP	regulation of angiotensin metabolic process	GO:0060177	0.04	*SUCNR1*
GO:BP	microtubule cytoskeleton organization involved in establishment of planar polarity	GO:0090176	0.04	*BRSK1*
GO:BP	carbohydrate homeostasis	GO:0033500	0.04	*BRSK1, SUCNR1*
GO:BP	glucose homeostasis	GO:0042593	0.04	*BRSK1, SUCNR1*
GO:BP	establishment of protein localization to extracellular region	GO:0035592	0.04	*BRSK1, SUCNR1*
GO:BP	protein secretion	GO:0009306	0.04	*BRSK1, SUCNR1*
GO:BP	renin secretion into blood stream	GO:0002001	0.04	*SUCNR1*
GO:BP	renal response to blood flow involved in circulatory renin-angiotensin regulation of systemic arterial blood pressure	GO:0001999	0.04	*SUCNR1*
GO:BP	protein localization to extracellular region	GO:0071692	0.04	*BRSK1, SUCNR1*

### Combined case-control GWAS

**Fig. 6:**
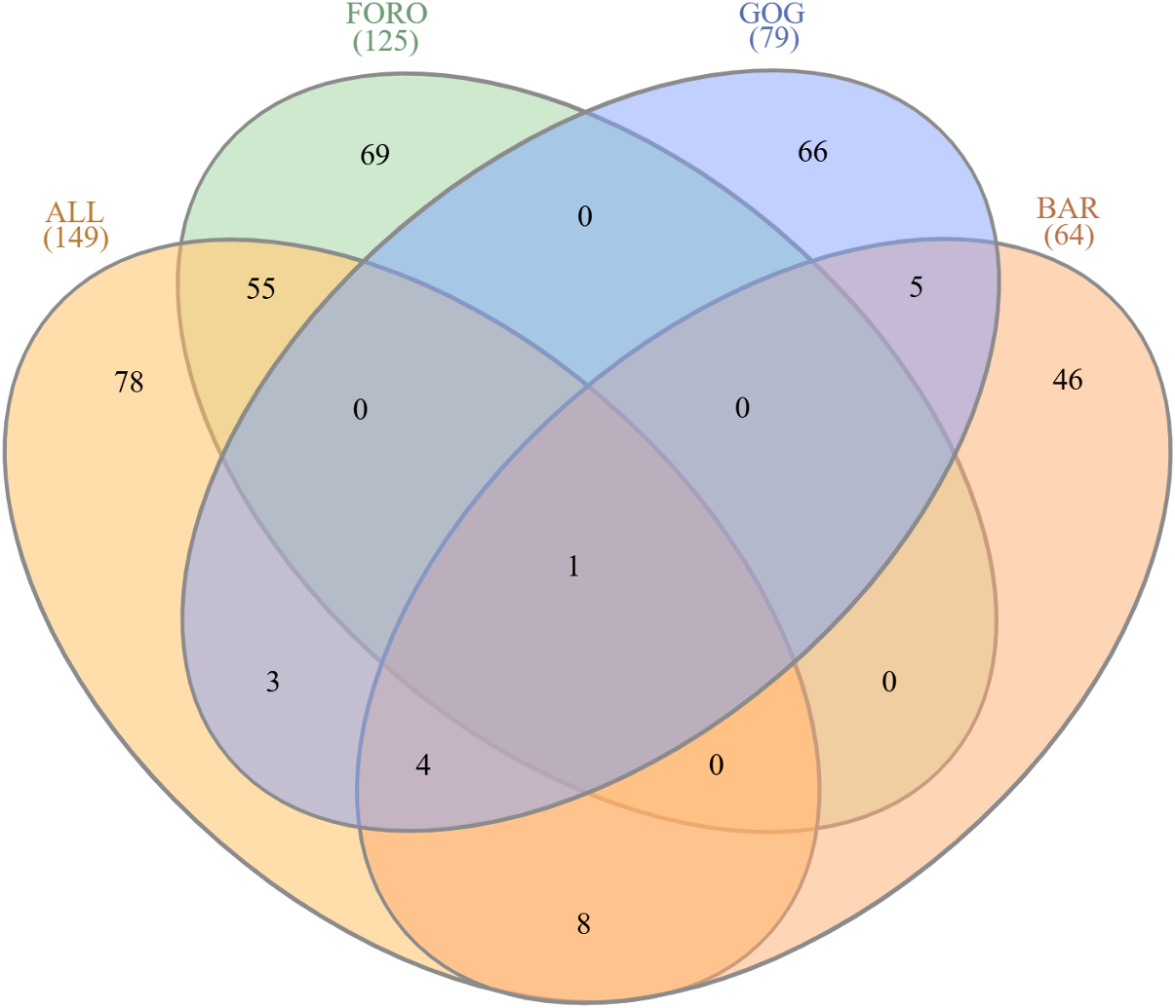
Venn diagram showing overlapping among candidate genes detected in different GWAS analyses; genes from the ALL dataset identified at suggestive significance and those from individual ecotype analyses identified at genome-wide significance.

No SNP reached genome-wide significance when BAR, FORO, and GOG were combined as a single case population and compared with the pooled controls (Supplementary Fig. 4) with a negligible genomic inflation (λ = 0.9514) (Supplementary Fig. 5). Several SNP exceeded the suggestive significance threshold and were located near the positional candidate genes *ASAP1, EIF2AK2*, and *FOXP1*.Genes located within 0.1 Mb of the suggestive SNP were annotated using the GALLO tool. Venn diagram analysis (Fig. 6) showed that *TH* (tyrosine hydroxylase) on chromosome 5 was the only gene common to all analyses, whereas *EIF2AK2* was shared across all ecotypes except FORO. Functional annotation identified a limited number of Gene Ontology categories associated with *TH* (Supplementary Material 1).

### Quantitative-traits GWAS

**Fig. 7:**
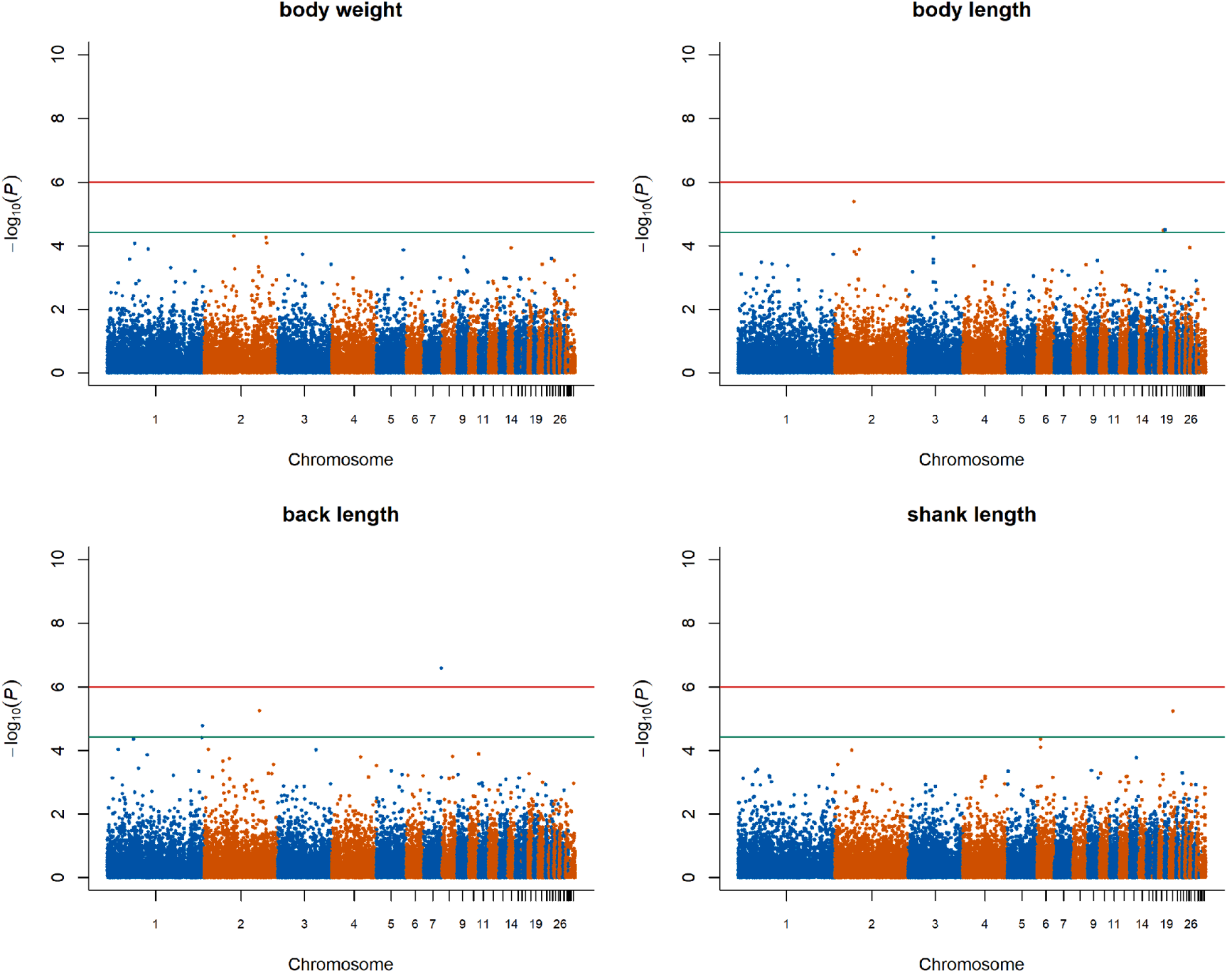
Manhattan plots of GWAS for body weight, body length, back length, and shank length performed in the eight Eritrean indigenous chicken ecotypes. Genome-wide and suggestive significance thresholds are shown by the red and blue lines, respectively.

Genome-wide association analyses were additionally performed using body weight, body length, back length, and shank length as quantitative phenotypes in the eight ecotypes included in the study (Fig. 7). No genome-wide significant associations were detected for body weight, body length, or shank length. A single SNP located on chromosome 7 (35.34 Mb) exceeded the significance threshold for back length and mapped within *CACNB4* gene, whereas no additional loci reached genome-wide significance for any of the analysed traits. Quantile–Quantile plots showed no evidence of inflation of the association statistics, with genomic inflation factors ranging from 0.987 to 1.005 (Supplementary Fig. 5).

## Discussion

The GWAS conducted on indigenous Eritrean chicken ecotypes provides new insights into the genetic basis of reduced body size, a trait potentially relevant to adaptation in resource-limited village chicken production systems (Abdi-Soojeede, 2022; Garces, 2000). This investigation compared three putative short-bodied ecotypes, BAR, FORO, and GOG, against a control cohort comprising the heaviest ecotypes, FM, ADTEK, HAYL, DEKE, and KEIH, using phenotypic metrics including back length, body weight, body length, and shank length.

Population structure analyses revealed extensive overlap among the eight ecotypes included in the GWAS, with weak differentiation observed in both PCA and ADMIXTURE analyses. This pattern is consistent with the management of African indigenous chickens as village ecotypes, where unrestricted gene flow and the absence of structured breeding programmes limit genetic subdivision among populations (Mwacharo et al., 2011; Lyimo et al., 2014). Consequently, the genomic regions identified in the case–control GWAS are unlikely to reflect exclusively genome-wide differentiation among ecotypes. Additional GWAS analyses using body weight, body length, back length and shank length as quantitative traits did not identify genome-wide significant associations, except for one back-length locus. Considering the limited sample size and the polygenic architecture of body-size traits, this result is not unexpected, because quantitative GWAS generally require substantially larger populations to detect loci with small or moderate effects (Visscher et al., 2017; Tam et al., 2019). Genomic inflation factors close to one further indicated adequate control of population stratification. Together, these results indicate that case–control associations are unlikely to be explained solely by population differentiation, although validation in larger populations is required to confirm their relationship with reduced body size.

Significant SNP were distributed across chromosomes 1, 2, 3, 4, 5, 7, 9, 12, 20, and 33 with the strongest signal observed on chromosome 3. This broad distribution, is consistent with recent chicken genomic studies reporting multiple population and trait-specific body-size loci (Wang et al., 2024; Luo et al., 2025; Nawaz et al., 2025; Wu et al., 2021). Nawaz et al. (2025) and Wu et al. (2021)suggests that body size traits are influenced by multiple genomic regions rather than a single major locus, further supporting a polygenic architecture.

Several associated regions were detected on chromosome 3 across the ecotype-specific analyses. However, these signals occurred at distinct genomic positions and should not be interpreted as evidence of a common underlying QTL.

Additional significant SNP on chromosomes 2 and 4 have likewise been linked to body composition, feed efficiency and skeletal development in broiler and indigenous populations (Raymond et al., 2018; Wang et al., 2024; Ye et al., 2025). This pattern mirrors large-scale genomic studies that identified numerous variants of small to moderate-effect influencing body weight and carcass traits (Dadousis et al., 2021).

### FORO ecotype

Among the positional candidate genes identified in the FORO ecotype, *TNS3* represents one of the most biologically plausible candidates because of its role in skeletal development and growth. *TNS3* regulates cytoskeletal organization and growth processes, and has been associated with fibular bone length in Pekin ducks, where QTL for body weight and breast muscle traits map to this locus (Deng et al., 2019). Evidence across species also links *TNS3* to osteogenic processes, skeletal development, and growth- plate cell proliferation (Zhang et al., 2023; Zhou et al., 2025). *TNS3* also exhibits signatures of positive selection in Ethiopian village chickens, linked to adaptation to high altitude, extreme temperatures, water scarcity, and limited feed (Vallejo-Trujillo, 2021). These observations support *TNS3* as a candidate for reduced body size of FORO, consistent with its conserved roles in bone development and cellular architecture across avian species.

*FOXP1* was also identified among the positional candidate genes. Previous studies have associated *FOXP1* with osteogenic differentiation and morphometric traits in livestock (Lan et al., 2023), supporting its potential role in skeletal development.

*CDK6* was associated with cell-cycle regulation, a biological process directly involved in tissue growth and development. Variants in this gene have previously been associated with morphometric traits in livestock and disease resistance in indigenous chickens (Grossel and Hinds, 2006; Xu et al., 2023; Liu et al., 2024).

*BDNF* was another positional candidate identified in the FORO ecotype. Besides its well-known role in neuronal development, *BDNF* has also been implicated in energy balance and growth regulation in vertebrates (Harvey and Rios, 2024). However, the functional enrichment analyses were supported by a limited number of genes and should therefore be interpreted with caution. The relatively small number of associated loci identified in FORO may reflect both the limited sample size and the complex polygenic architecture of body-size traits.

### GOG ecotype

The *CAPN1* gene emerged as a candidate in both combined (ALL) and GOG-specific analyses. Encoding the catalytic subunit of μ-calpain, it is central to postmortem protein degradation and meat tenderness (Foggi et al., 2021). In chickens, *CAPN1* gene polymorphisms are associated with growth rate and carcass traits (Rotwein, 2018).

The strongest association signals in GOG were detected on chromosome 3 near *EIF2AK2*, together with neighbouring genes including *RECK, MDH1*, and *UGP2*. The *EIF2AK2* gene is involved in the integrated stress response and regulation of protein synthesis (Bond et al., 2020; Taniuchi et al., 2016), whereas *MDH1* and *UGP2* participate in cellular energy metabolism. These genes represent plausible positional candidates, although their direct involvement in body-size regulation remains to be demonstrated.

Additional associations were detected near *FGF13* on chromosome 4. Members of the fibroblast growth factor family are known regulators of skeletal development, and *FGF13* has previously been associated with growth-related traits in livestock (Cai et al., 2024). Although functional enrichment suggested the involvement of growth- and metabolism-related biological processes, these categories were supported by a limited number of genes and should therefore be cautiously interpreted with caution.

### BAR ecotype

The *IGF2* gene appeared as a suggestive candidate in ALL and BAR analyses, supporting a polygenic basis for size variation. The sub-genome-wide peak near *HMGA2* on chromosome 1 is notable, given its established role in chicken dwarfism via pituitary hypoplasia and somatotroph dysfunction (Perini et al., 2023; Zhou, 2021). Although the signal did not reach the Bonferroni-corrected threshold, its proximity to a known stature QTL, together with the chromosome-5 *IGF2* association, strengthens its candidacy. The IGF2 promotes foetal and postnatal growth via *IGF1R* signalling, with imprinted variants explaining 10–15% of body weight variance in chickens (Wu et al., 2026). In birds, *IGF2* influences bone and muscle development, post-hatching growth, and feather development (Guo et al., 2023; Rotwein, 2018; Wang et al., 2026; Yan et al., 2025). It also modulates muscle hypertrophy, fat deposition, and feed efficiency across livestock (Wang et al., 2026). *HMGA2* directly regulates the RNA-binding protein *IGF2BP2*, which enhances *IGF2* translation (Li et al., 2012), and both genes lie within the same functional network (Nawaz et al., 2025).

*BRSK1* and *SUCNR1* were also located near associated regions. Both genes are involved in cellular metabolism and signalling (Bendzunas et al., 2024; Ariza et al., 2012).

### Combined and quantitative-trait GWAS

Combining the putative short-bodied ecotypes yielded no genome-wide significant variants, indicating genetically distinct causal variants across ecotypes. Suggestive SNP on chromosome 1 mapped near *ASAP1* gene, a GAP (GTPase-activating protein) that negatively regulates Arf (ADP ribosylation factor) proteins involved in embryonic development and growth (Liu et al., 2024; Xi et al., 2023).

Complementary GWAS analyses using body weight, body length, back length, and shank length as quantitative traits across the eight ecotypes were also performed. No genome-wide significant associations were detected for body weight, body length, or shank length, whereas a single significant SNP on chromosome 7 was identified for back length, mapping within *CACNB4* gene that mediates calcium ion influx, modulates calcium channel voltage, and regulates gene transcription (Wang et al., 2025). The limited number of significant associations is consistent with the relatively small sample size and the polygenic architecture of body-size traits, which reduce the power to detect variants with small or moderate effects. Genomic inflation factors close to one indicated adequate calibration of the association models and limited residual inflation due to population structure.

Venn-diagram analysis identified *TH* as the only positional candidate gene shared among the three ecotype-specific analyses and the combined GWAS. *TH* is located approximately 25 kb from *IGF2* on chromosome 5 and encodes the rate-limiting enzyme in catecholamine biosynthesis (Carrier et al., 1993). Although its repeated identification across analyses may indicate a region of interest, positional overlap alone does not demonstrate a causal role in reduced body size and requires further validation.

Several positional candidate genes identified in this study have previously been associated with growth-related traits in other livestock species. For example, *HMGA2* and *IGF2* have consistently been associated with body size in chickens (Perini et al., 2023), whereas *FGF13* has been implicated in skeletal growth in cattle (Cai et al., 2024).

These observations support the biological plausibility of the identified candidate genes, although functional validation is required to confirm their role in reduced body size in Eritrean indigenous chickens. Recent chicken studies likewise show that body-size-associated loci vary among populations, although recurrent regions on chromosomes 1 and 4 include *HMGA2, LCORL, LDB2, NCAPG*, and *IGF2BP1* (Perini et al., 2023; Wang et al., 2024; Jiang et al., 2025; Sun et al., 2026). This partial recurrence alongside extensive population-specific signals is consistent with the ecotype-specific pattern observed in this study.

These findings are consistent with the polygenic architecture of body-size traits and the limited statistical power expected in relatively small populations, despite adequate control of population stratification by the mixed linear model.

This study identified candidate genomic regions associated with reduced body size in Eritrean indigenous chicken ecotypes. Several biologically plausible candidate genes, including *HMGA2, IGF2, TNS3, FGF13, CAPN1*, and *EIF2AK2*, were located near associated genomic regions and represent promising targets for future investigation. However, the present study relied exclusively on morphometric measurements to define putative short-bodied ecotypes; the association should therefore not be interpreted as definitive evidence of genetic dwarfism or causality. Recent genomic and transcriptomic studies further indicate that reduced body size can a rise through heterogeneous, breed-specific mechanisms (Wu et al., 2026; Sun et al., 2026; Haunshi et al., 2025). Future studies using larger populations, independent validation datasets, and functional approaches will be necessary to confirm the biological relevance of these loci and to better understand the genetic basis of body-size variation in African indigenous chickens.

## CRediT authorship contribution statement

**Hortuma A. Habteslasie:** Writing – review & editing, Writing – original draft, Validation, Methodology, Formal analysis, Data curation, Conceptualization. **Francesco Perini:** Writing – review & editing, Writing – original draft, Visualization, Validation, Software, Methodology, Formal analysis, Data curation, Conceptualization. **Kiplangat Ngeno:** Writing – review & editing, Supervision, Software, Methodology, Formal analysis. **Daniele Colombi:** Writing – review & editing, Visualization, Validation, Formal analysis, Data curation. **Emiliano Lasagna:** Writing – review & editing, Validation, Supervision, Project administration, Funding acquisition. **Tadelle Dessie:** Writing – review & editing, Supervision, Resources. **Alexander Kahi:** Writing – review & editing, Supervision, Resources, Conceptualization.

## Disclosures

The authors declare that they have no known competing financial interests or personal relationships that could have appeared to influence the work reported in this paper.

## Appendix Supplementary materials

Image, application 1

Image, application 2

## Footnotes

Supplementary material associated with this article can be found, in the online version, at doi:10.1016/j.psj.2026.107502.
