# Supplementary material for: Genetic variants associated with reduced body size in indigenous chickens of Eritrea: A genome-wide association study
Source: Poult Sci. 2026 Jul 27;105(11):107502. doi: 10.1016/j.psj.2026.107502 (PMC13487389; doi:10.1016/j.psj.2026.107502)
Supplement: Supplementary file 1 [file mmc1.docx]

**SUPPLEMENTARY FIGURES**

**Supplementary Figure 1.** Quantile–Quantile (Q–Q) plot of the genome-wide association analysis performed for the FORO ecotype.

**Supplementary Figure 2.** Quantile–Quantile (Q–Q) plot of the genome-wide association analysis performed for the GOG ecotype.

**Supplementary Figure 3.** Quantile–Quantile (Q–Q) plot of the genome-wide association analysis performed for the BAR ecotype.

**Supplementary Figure 4.** Manhattan plot of the combined GWAS comparing all putative short-bodied ecotypes with the control population. The blue horizontal line indicates the suggestive significance threshold, whereas the red horizontal line represents the genome-wide significance threshold.

**Supplementary Figure 5.** Quantile–Quantile (Q–Q) plot of the combined genome-wide association analysis performed using all putative short-bodied ecotypes against the control population.

**Supplementary Figure 6.** Quantile–Quantile (Q–Q) plots of genome-wide association analyses performed for body weight, body length, back length, and shank length using quantitative phenotypes.
